# New Insights into Antithrombotic Therapy for Cardio- and Cerebrovascular Disease: From Molecular Mechanisms to Clinical Application

**DOI:** 10.3390/jcdd10060258

**Published:** 2023-06-14

**Authors:** Plinio Cirillo, Giovanni Cimmino

**Affiliations:** 1 Department of Advanced Biomedical Sciences, University of Naples Federico II, Via S. Pansini 5, 80131 Naples, Italy; 2 Department of Translational Medical Sciences, University of Campania Luigi Vanvitelli, Via L. Bianchi, 80131 Naples, Italy; giovanni.cimmino@unicampania.it

Thrombosis has a pivotal role in the pathophysiology of acute cardiovascular events such as myocardial infarction and stroke [1]. Thus, an impressive effort has been done to better understand all those mechanisms able to promote intravascular thrombosis, in order to draw a better antithrombotic strategy to avoid future events. This Special Issue, by collecting the points of view of authoritative international research groups, gives an updated overview of the state-of-the-art as well as of the most promising future prospects for mechanisms of thrombosis and antithrombotic therapy.

The first article published comes from Prof Angiolillo’s group [2]. It explores the role of antiplatelet monotherapy with P2Y12 inhibitors in patients undergoing percutaneous revascularization. By critically analyzing the available literature, the Researchers conclude that monotherapy with P2Y12 inhibitors is a current possibility that however needs to be better evaluated in well-designed clinical trials to carefully establish its onset after a period of DAPT. This paper underscores that antiplatelet monotherapy with a P2Y12 inhibitor has been already considered a reasonable antiplatelet strategy by the European and American guidelines in patients treated with PCI. Moreover, it gives complete information about the RCTs specifically designed to obtain new insights for P2Y12 inhibitor monotherapy even in patients with STEMI, and for long-term secondary prevention in patients with stable coronary artery disease.

Another historical actor on the stage of antiplatelet therapy is faced in the paper by Prof Cattaneo’s group [3], who focuses on aspirin monotherapy, concluding that the enteric-coated formulation is less effective in terms of antiplatelet activity, especially in patients with more than 70 kg of weight. Therefore, by considering its more favorable pharmacological profile, plain aspirin should be the preferred formulation for cardiovascular prevention [3].

An important take-home message comes from the paper of Prof Davlouros’ group [4]. Specifically, the Authors deal with a patient typology usually excluded by clinical trials, represented by cancer patients, who are at high risk of both ischemic and bleeding events. The high ischemic/bleeding risk of oncologic patients, due to the dysregulation of their hemostatic system by cancer, requires an appropriate duration and optimal antiplatelet therapy after PCI and/or acute coronary syndromes not treated with stent implantation. The use of new-generation DES may lead to shortened DAPT duration in all-comer patients, including patients with cancer. Current guidelines indicate that the optimal duration of DAPT should be 1–3 months, consisting of aspirin and clopidogrel, while TAT, if required, might only be administered for a short period of time (up to 1 week in the hospital), followed by a DOAC and a single oral antiplatelet agent (preferably clopidogrel). The advancements in other structural interventions, such as TAVR, PFO-ASD closure, and LAA occlusion, and non-cardiac diseases, such as PAD and CVA, may require DAPT, thus it is indisputable that a multidisciplinary approach is necessary for this to better balance thrombotic and bleeding risk [4].

Of course, antiplatelet therapy represents only one of the two faces of antithrombotic therapy since the coagulation pathway is involved in the pathophysiology of thrombosis too [5,6]. Thus, of extreme interest is the report by Prof. J. Badimon et al. that analyzes in detail the inhibition of factor XI/XIa as an attractive future option as antithrombotic therapy [7]. A better understanding of the contact pathway, especially of its significant role in thrombus stabilization and growth vs. in the initiation of clot formation, has opened up new targets for therapeutic intervention. FXI is one such promising target. FXI-directed strategies could offer similar protection against thrombotic events as DOACs, but with the advantage of lower bleeding risk. Growing strategies are now available to modulate FXI/FXIa, including ASOs, small molecules, antibodies, and aptamers. A wide variety of clinical scenarios may take advantage of this novel strategy. Several FXI-directed agents are currently undergoing clinical evaluations in phase II and phase III trials [7].

In line with this view on the future, the article proposed by Prof. G. Vilahur first comments on the key limitation of the currently used antithrombotic regimes in ischemic heart disease and ischemic stroke and then it looks at the emerging anticoagulant and antiplatelet agents in the pipeline with the potential to improve clinical outcomes. Some of these promising strategies are still under biological evaluation, others have reached the animal model test, and only few agents have been considered for randomized clinical trials [8].

Although many studies have clearly indicated that advantages of DOAC to prevent thrombotic events in specific clinical settings, some grey areas still exist when DOAC is compared to VKA [9]. This is the case of intracardiac thrombosis. On this important issue are focused the articles by Prof Pradhan [10] and Prof Chan [11]. Specifically, Pradhan et al. face the left ventricular thrombosis after myocardial infarction by critically analyzing the available literature including case reports, small trials, and meta-analysis, and supports the use of DOACs over VKA. Interestingly, an algorithm for the choice of agent and duration of the strategy has been also proposed [10]. On the other hand, the meta-analysis by Prof Chan’s group on left atrial appendage thrombosis, which is main cause of cardioembolism and ischemic stroke, strengthens the role of DOACs also in this clinical context. Targeted clinical studies are warranted to better define its use in clinical practice to help clinicians in the choice of DOAC and duration of treatment [11].

Current guidelines recommend P2Y12 inhibitors plus aspirin as the gold standard of antiplatelet therapy in ACS and PCI-treated patients [12]. However, the onset of action for P2Y12 inhibitors is about one hour after administration [13]. Cangrelor, an injective P2Y12 inhibitor, by having an immediate effect on platelet aggregation, represents a new, extremely attractive therapeutic horizon in the use of parenteral P2Y12 inhibitors. Prof Andò and his research group analyzed the available data on the current use and potential of this new strategy [14]. The currently available oral P2Y12 have a relatively slow onset of action, so drug-naïve patients, and especially those with ACS, undergoing PCI lack of antiplatelet protection in the first hours after oral administration of antiplatelet therapy, thus the patients may be exposed to a greater thrombotic risk. Cangrelor might overcome this gap. Its effectiveness in drug-naïve patients undergoing PCI has been proved, both in the stable and the acute setting, by reducing early and 30-day ischemic outcomes, with particular emphasis on ischemia driven revascularization and early ST. Cangrelor appears to be a very safe drug with a low rate of bleeding and specifically of major events [14]. In this regard pharmacological research will open shortly a new debate about the optimal choice and timing to administer parenteral DAPT, since a new drug to be self-administered at home (selatogrel), or in the ambulance (zalunfiban) will be available in parallel to a drug to be administered in the hospital (cangrelor). Further RCTs are needed about the combination of parenteral and potent oral P2Y12 inhibitors in patients with ACS and about the optimal switching strategies.

Patients with peripheral artery disease have often been considered the Cinderella of patients with atherosclerosis in terms of thrombosis risk. Thus, the importance of antithrombotic therapy has been underestimated. The article by Prof. M. Bonaca et al. provides an overview of current evidence in different clinical settings in PAD and proposes an algorithm for antithrombotic therapy management in daily practice [15]. In patients undergoing revascularization, evidence supports more aggressive antithrombotic therapy, specifically, dual pathway inhibition after low extremity revascularization, irrespective of the type of intervention. The optimal management of patients undergoing revascularization for carotid and abdominal aortic disease remains to be better elucidated. The development of newer antithrombotic strategies, such as factor XIa inhibitors, may play an important role in this regard [15].

Finally, to complete the Special Issue, we find an original article from Prof. Xiao’s research group in which the Authors provided further basic evidence on the role of metformin in stabilizing atherosclerotic plaque by binding MMP-9 and driving its degradation thus preserving the collagen content of plaque and improving atherosclerotic plaque stability [16]. Metformin remains a drug of choice for the treatment of type 2 diabetes with proven glucose-lowering effectiveness, safety, favorable effect on body weight, and low cost. Beyond these properties, an atherosclerotic stabilizing effect is here expanded and added to the known anti-inflammatory activity [16].

In summary, since the role of platelets and of the coagulation cascade in the pathophysiology of cardiovascular thrombosis have been extensively investigated, the research on antithrombotic options is extremely active and in progress. A more appropriate use of drugs already in use and the search for new safer drugs in terms of ischemic and bleeding risk might be considered a glimpse into the future to which this special issue contributes in an important way by acting as a hypothetical user manual.
